# Social Relationships and Self-Perceptions of Aging

**DOI:** 10.1093/geroni/igaa057.1456

**Published:** 2020-12-16

**Authors:** Hanamori Skoblow, Christine Proulx

**Affiliations:** University of Missouri, Columbia, Missouri, United States

## Abstract

Recent research has demonstrated that social relationships are positively associated with self-perceptions of aging (SPA; Santini et al., 2019), although to date, this evidence is cross-sectional. The current study builds on previous work and explores the longitudinal relation between social relationships and SPA, and whether these associations might be buffered by perceived mastery. Using repeated measures data from three waves (2008, 2012, and 2016) of the Health and Retirement Study (HRS), we examined how relationship quality (i.e., support and strain) influenced self-perceptions of aging among adults aged 65+ (n = 1477). Greater support from friends in 2008 was significantly associated with better SPA in 2016, and this effect was amplified by high levels of mastery in 2012. That is, older adults with high mastery and high friend support reported more positive SPA than individuals with less mastery or friend support, controlling for gender, age, race, education, income, depressive symptoms, self-rated health, and baseline SPA. Relationship quality with spouse, children, and other family members were not significant predictors of SPA, nor did mastery moderate the association between these relationships and SPA. These results provide evidence for the importance of interpersonal factors such as friendship quality and individual factors in understanding older adults’ perceptions of the aging process.

